# Immortalized Mesenchymal Stem Cells: A Safe Cell Source for Cellular or Cell Membrane-Based Treatment of Glioma

**DOI:** 10.1155/2022/6430565

**Published:** 2022-04-13

**Authors:** Yuxuan Zhang, Jie Liu, Yunzhao Mo, Zetao Chen, Taoliang Chen, Yan Li, Yaofeng Zheng, Shaokang Deng, Xiangdong Xu, Huajian Chen, Haoqi He, Jiansheng Chen, Tao Jin, Xinlin Sun, Yiquan Ke, Jihui Wang

**Affiliations:** ^1^The National Key Clinical Specialty, The Engineering Technology Research Center of Education Ministry of China, Guangdong Provincial Key Laboratory on Brain Function Repair and Regeneration, Department of Neurosurgery, Zhujiang Hospital, Southern Medical University, Guangzhou 510282, China; ^2^Department of Neurosurgery, Shantou Central Hospital, Affiliated Shantou Hospital of Sun Yat-sen University, Shantou 515041, China

## Abstract

Mesenchymal stem cells (MSCs) have emerged as putative therapeutic tools due to their intrinsic tumor tropism, and anti-tumor and immunoregulatory properties. The limited passage and self-differentiation abilities of MSCs in vitro hinder preclinical studies on them. In this study, we focused on the safety of immortalized mesenchymal stem cells (im-MSCs) and, for the first time, studied the feasibility of im-MSCs as candidates for the treatment of glioma. The im-MSCs were constructed by lentiviral transfection of genes. The proliferative capacity of im-MSCs and the proliferative phenotype of MSCs and MSCs co-cultured with glioma cells (U87) were measured using CCK-8 or EdU assays. After long-term culture, karyotyping of im-MSCs was conducted. The tumorigenicity of engineered MSCs was evaluated using soft agar cloning assays. Next, the engineered cells were injected into the brain of female BALB/c nude mice. Finally, the cell membranes of im-MSCs were labeled with DiO or DiR to detect their ability to be taken up by glioma cells and target in situ gliomas using the IVIS system. Engineered cells retained the immunophenotype of MSC; im-MSCs maintained the ability to differentiate into mesenchymal lineages in vitro; and im-MSCs showed stronger proliferative capacity than unengineered MSCs but without colony formation in soft agar, no tumorigenicity in the brain, and normal chromosomes. MSCs or im-MSCs co-cultured with U87 cells showed enhanced proliferation ability, but did not show malignant characteristics in vitro. Immortalized cells continued to express homing molecules. The cell membranes of im-MSCs were taken up by glioma cells and targeted in situ gliomas in vivo, suggesting that im-MSCs and their plasma membranes can be used as natural drug carriers for targeting gliomas, and providing a safe, adequate, quality-controlled, and continuous source for the treatment of gliomas based on whole-cell or cell membrane carriers.

## 1. Introduction

Tumors, especially some refractory tumors, such as glioma, are a commonly encountered clinical challenge in the contemporary scenario. Traditional treatments for glioma include surgery, radiotherapy, and chemotherapy, but the prognosis is still poor [1]. Current efforts are focused on developing targeted molecular therapies, immunotherapies, gene therapies, and novel drug delivery technologies [2]. Cell-based active targeted drug delivery is advantageous because of its inherent targeting capabilities, biocompatibility, and host integration, providing therapeutic efficacy and minimal side effects [3]. Natural drug carriers, such as cells and cell membranes, are very promising candidates for treating tumors [4]. Or the artificial synthetic carries are modified by the specific cell membranes to realize the biomimetic drug carrier to treat tumors [5]. They appear to be complex but can take advantage of the inherent tissue targeting capabilities of specific cell types due to ligand-receptor binding and specific chemokines to achieve actively targeted drug delivery [3], such as CXCR4 and SELP, and SELPLG. Stem cells are known to have self-renewal and differentiation abilities. Furthermore, in recent years, studies have suggested that certain stem cells also have tumor-homing ability [6]. Mesenchymal stem cells (MSCs) are adult stem cells isolated from various tissues, such as the umbilical cord [7], adipose tissues [8], and bone marrow [9]. Owing to the tumor tropism of stem cells, they are potential candidates for anti-tumor therapy either by carrying drugs or by secreting anti-tumor agents. For instance, engineered mNSCs secrete tumor necrosis factor apoptosis-inducing ligand to the tumor cavity of glioblastoma (GBM)-bearing mice [10]. However, limited passage ability and cell senescence during in vitro culture limit the applicability of MSCs as a long-term cellular source for tumor therapy. Furthermore, different individuals and tissue-derived MSCs are heterogeneous [11].

Additionally, there are different functional subsets of MSCs from the same organism. It is necessary to immortalize MSCs to obtain a stable, sufficient, and homogeneous cell source or long-acting therapeutic cell. MSCs have an anti-tumor ability from specific sources. In a previous study, Tátrai et al. constructed an immortalized cell line by transfecting a combination of BMI1 and TERT to human adipose-derived MSCs (hADSCs) without significantly perturbing their phenotype or biological behavior [12]. In our study, we used this method to obtain two types of immortalized MSCs (im-MSCs), human umbilical cord MSCs (hUCMSCs) and hADSCs. In previous studies, it was considered that im-MSCs were not tumorigenic [13]; however, establishing the safety of im-MSCs still requires more clinical research. As a drug carrier, MSC membranes are safer and more biocompatible than the whole MSCs. The plasma membrane is the main functional part in the homing ability of MSCs. The tumor trophic characteristics of MSCs are mainly attributed to the receptor and transmembrane proteins on the membrane. The key ligands involved in MSC homing include chemokine receptors CCR2, CXCL12, and CXCR4 and adhesion ligands, such as SELPLG and SELP, among which CXCR4 is one of the most important molecules mediating the tumor-homing effects of MSCs [14].

We constructed two representative immortalized human MSCs (hMSCs) that overexpressed the TERT gene and BMI1 gene in the present study. The immunophenotype and directed differentiation ability of the cells before and after transfection were examined. The karyotype of immortalized cells was analyzed after long-term culture to understand the gene stability. Furthermore, we thoroughly evaluated the tumorigenicity of im-MSCs in both in vitro and in vivo, especially in the brain. We also determined the safety of im-MSCs in the treatment of brain tumors, especially glioma, as a cellular drug carrier. Furthermore, we extracted the cell membranes of the im-MSCs and detected their ability to be taken up by glioma cells. Lastly, we tested MSC membranes targeting in situ gliomas to understand their potential, including as drug carriers.

## 2. Materials and Methods

### 2.1. Cell Isolation and Culture

hUCMSCs were isolated from Wharton's jelly of human umbilical cord by tissue explants method [7]. hUCMSCs were cultured in Human Umbilical cord mesenchymal stem cell Basal Medium (Cyagen Biosciences Inc., USA) containing 10% fetal bovine serum (Cyagen Biosciences Inc., USA). hADSCs were cultured in Human adipose mesenchymal stem cell Basal Medium (Cyagen Biosciences Inc., USA) containing 10% fetal bovine serum (Cyagen Biosciences Inc., USA), which were a generous gift from Pro. Hongbo Guo (Southern Medical University, China).

The human GBM cell lines U87 (American Type Culture Collection, USA) and U251 (China Academia Sinica Cell Repository, China) were cultured in Dulbecco's modified Eagle's medium (DMEM) containing 10% fetal bovine serum [15] (Biological Industries, Israel). HAs (ScienCell Research Laboratories, USA) and HEB were grown in 1640 (Gibco, CA, USA) containing 10% fetal bovine serum (Biological Industries, Israel). The U87-Luc cell line was generated in our laboratory via transfection with a reporter gene encoding firefly luciferase. All cells were grown in a humidified atmosphere of 5% CO_2_ at 37°C.

### 2.2. Immunophenotyping

MSCs were cultured to P5, and im-MSCs were cultured to P10. Cells were isolated with 0.25% trypsin that contain 0.1% ethylenediaminetetraacetic acid and washed by PBS (Gibco, CA, USA) three times. The MSCs were cultured with CD11b (PE), CD14 (APC), CD29 (PE), CD31 (FITC), CD34 (PE), CD44 (FITC), CD45 (FITC), CD73 (APC), CD90 (PE), and CD105 (FITC) monoclonal antibodies for 20 minutes at RT, and detected with a BD FACSVerse flow cytometer. The data were analyzed by FlowJo software (BD, USA).

### 2.3. Human Umbilical Vein Endothelial Cells (HUVEC) Tube Formation Assay

After the hMSCs (P10) and the im-hMSCs (P10) adhere to the T25 culture flask, aspirate the original complete medium, wash 3 times with PBS to eliminate the influence of the serum in the original medium, add ECM basal medium to culture for 48 hours, and collect the medium. HUVECs cultured in the aforementioned conditioned medium for 24 hours.

Matrigel (BD Biosciences, USA) was prepared and kept on ice until used. 100 *μ*l Matrigel was added to the wells of 24-well plate evenly and incubated at 37°C for 30 min. HUVECs (1.5 × 10^5^) were incubated in 500 *μ*l basal medium for 4 h before image taking. The capillary tubes were quantified under a 100x bright-field microscope, by measuring the total numbers of the completed tubule structure [16].

### 2.4. Isolation of PBMCs

Peripheral blood mononuclear cells (PBMCs) were obtained from the peripheral blood of healthy volunteers. Briefly, carefully layer 5.0 ml of anti-coagulated whole blood over 5.0 ml of Ficoll lymphatic separation fluid in a 15 ml centrifuge tube [17]. Centrifuge the tubes at 500 g for 30 minutes in a swing-out rotor at 20°C. Allow the rotor to decelerate without the break. After centrifugation, a white band at the sample/medium interface is PBMCs. This band was harvested in a 15 ml tube and the cells pelleted at 400 g for 10 min (18-22°C). They are resuspended in saline or culture medium, sedimented again, and then resuspended in a medium compatible with the subsequent process.

### 2.5. PBMC Proliferation Assay

Seed 1.5 × 10^5^ hMSCs (P10) or im-hMSCs (P10) in a 12-well plate. After the cells adhered, they were treated with a complete medium containing mitomycin C (10 mg/mL, Roche) for two hours. After washing the cells 3 times with D-PBS, add PBMCs to each well at a ratio of 5 : 1 (PBMCs: MSCs). The PBMCs were incubated with phytohemagglutinin L (PHA L) (10 mg/mL, ThermoFisher) for 3 days in the absence or presence of hMSCs [17]. The PBMCs in each well were collected and resuspended in an equal volume of medium, and the corresponding amount of CCK-8 reagent was added to spread on the 96-well plate. Repeat 3 times for each group. After incubating for 2-4 hours, use the microplate reader to detect the OD450 value of each group of cells.

### 2.6. Lentiviral Gene Transfer

LV-TERT (pLOV-EF1a-PuroR-CMV-TERT-3FLAG) were purchased from Genechem (Shanghai, China). LV-BMI1 (pLOV-EF1a-NEO-CMV-BMI1) were purchased from Genepharma (Suzhou, China). Cell culture using 500 *μ*l of polybrene-containing (5 *μ*g/ml) medium in 24-well plates overexpressing lentivirus was transfected with primary MSCs at multiplicity of infection (MOI) of 50. Cells were selected and maintained with puromycin (5 *μ*g/mL, Solarbio, China) or G418 (500 *μ*g/ml, Macklin, Shanghai, China) after transfection for 72 hours.

HA1800 transfected using LV-mCherry-Luc (pLenti-CMV-mCherry-linker-Luc-PGK-Blasticidin, OBiO, Shanghai, China) at MOI of 1 was selected and maintained with Blasticidin (10 *μ*g/ml). hUCMSC-Luc, hADSC-Luc, im-hUCMSC-Luc, and im-hADSC-luc were also obtained by the above method, but different MOI of 50.

### 2.7. RNA Isolation and RT-PCR

Extraction of total RNA from hMSCs (P5, P10, P15, P20, P25) and im-hMSCs (P10, P15, P20, P25, P30, P35, P40, P45, P50) with TRIzol (Takara, Japan), and the concentration of RNA were measured by using NanoDrop2000 spectrophotometer (Thermo Fisher, USA). 1000 ng of RNA was reverse-transcribed into cDNA by using the PrimeScript™ RT Reagent Kit with gDNA Eraser (Takara, Japan). Quantitative RT-PCR was performed in a CFX96 real-time PCR detection system (Bio-Rad, USA) using SYBR premix (Takara, Japan). All reactions were repeated three times, and human GAPDH was the endogenous control. Primers for detecting gene expression are shown in Supplementary Table 1.

### 2.8. Western Blotting and Analysis of MSC Membrane Proteins

Western blotting was performed as previous research [18]. After hMSCs (P10) and im-hMSCs (P10) lysing with RIPA (CWBio, Beijing, China) on ice for half an hour, the supernatant was centrifuged to obtain total protein. After measuring the protein concentration by the BCA assay, the loading buffer was added to the supernatant and boiled for ten minutes to obtain protein samples. The protein sample was separated in SDS-PAGE gels and the target protein was transferred to the PDVF transfer membranes (Millipore, Germany) according to the guidelines of protein ladder (Thermo Fisher, USA) and the membranes were blocked with 5% BSA for 1 hour at RT. The membranes were incubated with the primary antibody overnight at 4°C, washed three times with TBST every ten minutes, and then incubated with the secondary antibody for 1 hour at room temperature (RT) before washing. Visualization of the immunoreactive band using Image Lab (Bio-Rad, USA) after adding luminol-based chemiluminescent substrate (ECL, Millipore, Germany). The band density was analyzed with Image Lab software (Bio-Rad, USA). The antibodies used for detecting proteins expression are shown in Supplementary Table 2.

### 2.9. Differentiation Studies In Vitro

Adipocytic Differentiation. MSCs (P10) and im-MSCs (P10) isolated from 6-well plate with trypsin-EDTA, 4 × 10^5^ cells were added to the new culture plate, and the medium consists of 1*μ*M dexamethasone, 10 mg/L insulin, and 0.5 mM IBMX, and 200*μ*M indomethacin was added after 24 hours of liquid exchange. The medium changed every third day during 2-3 weeks. MSCs were fixed in 4% paraformaldehyde after cells differentiation, stained with oil red O. Then, specimens were placed under a microscope (Leica, Germany) and photographed.

Osteogenic Differentiation. The cells were seeded in a new 6-well plate in the same way as above, cultured in medium that contain 0.1 *μ*M dexamethasone, 50 *μ*g/L VitC, and 10 mM beta-glycerophosphoric acid disodium salt. The differentiated cells were fixed with 4% paraformaldehyde and stained with ruthenium, recording with a microscope (Leica, Germany).

Chondroblastic Differentiation. MSCs (P10) and im-MSCs (P10) pellets containing 4 × 10^5^ cells were cultured in ChondroDiff Medium (Bgscience, Guangzhou, China) for 2-3 weeks. The medium changed every third day during the period. The medium consists of ITS supplement, TGF-*β*3, ascorbate, proline, dexamethasone, sodium pyruvate, penicillin, and streptomycin. The cell pellets were fixed in 4% paraformaldehyde and subjected to conventional paraffin sectioning. Sections were stained with alcian blue. The images were obtained with a microscope (Leica, Germany).

### 2.10. Cell Proliferation Assay

Cell counting Kit-8 (CCK8) and EdU assay were used to investigate proliferation. We used a CCK-8 kit (Dojindo, Shanghai, China) to measure proliferation of cells. MSCs and im-hMSCs were cultured to P10. Then, 100 *μ*l of cell suspension containing 1000 cells was added to each well in a 96-well plate and placed in a 37°C incubator containing 5% CO_2_. After the cells were attached, a mixture of 10 *μ*l of CCK-8 reagent and 90 *μ*l of medium was added to each well of a 96-well plate, and five replicate wells were repeated. Incubate in a 37°C incubator containing 5% CO_2_ for 1 to 2 hours, then test the OD450 with a microplate reader (Biotex, USA).

In addition, the cells were cultured in 96-well plates and treated with 100 *μ*l of medium containing 20 *μ*M EdU (5-ethynyl-20-deoxyuridine, Keygene, China). After incubating for 2 hours at 37°C containing 5% CO_2_, the cells were fixed with 4% paraformaldehyde for 15 minutes. Cell nuclei were stained with DAPI. The rate of proliferation was calculated according to the manufacturer's instructions (KeyFluor488 Click-iT EdU Kit, keygen BioTECH, Jiangsu, China). Finally, images were obtained with an inverted fluorescence microscope (Leica, Germany).

### 2.11. Soft Agar Assay

1.2% argrose (Macklin, Shanghai, China) solution and 2× medium were mixed in a ratio of 1 : 1, and 1.5 ml of the mixture was quickly added to each well of a 6-well plate, and the gel was allowed to solidify at RT. Then, 0.7% argrose and 2× medium were mixed in 1 : 1 ratio, and cell (P10) suspension of 10,000 cells per well was added to the above mixture. The mixture was quickly added to 6-well plate at 1 ml per well after mixing. After the upper agar was solidified, the plate was placed in 37°C incubator with 5% CO_2_ for 2-3 weeks. Repeat three wells for each cell line.

### 2.12. Safety Study In Vivo

Human umbilical cord mesenchymal stem cells (P15) and im-hUCMSCs (P15) carrying the luciferase gene were injected intracranially or subcutaneously into 4-week-old female BALB/c nude mice (Medical Laboratory Animal Center of Southern Medical University, China). After intraperitoneal injection of fluorescein substrate for 10 minutes, in vivo imaging system (IVIS, Lumina II, USA) was used to detect intracellular growth of the cells for 4, 7, and 14 days. U87-Luc was used as a positive reference. HA1800-Luc was used as a negative reference.

### 2.13. Isolation of MSC Plasma Membranes

Separation of the plasma membranes (PM) from MSCs (P15) by previous methods [19]. Briefly, the cell pellet isolated from the culture dishes were resuspended in IB-1 buffer (225 mM mannitol, 75 mM sucrose, 30 mM Tris-HCl, 0.5% BSA, 0.5 mM EGTA, protease inhibitor cocktail, phosphatase inhibitor cocktail, pH 7.4) and the cells were lysed with an ultrasonic breaker, and then centrifuged at 800*g* for 5 mins at 4°C to discard the pellet, repeat the previous step to remove nuclei and unbroken cells. Continue to separate the supernatant at 10000*g* for 5 mins at 4°C with a centrifuge and discard the pellet. And then centrifuged at 25000*g* for 20 mins at 4°C discard the supernatant. The final pellet was dissolved in IB-2 buffer (225 mM mannitol, 75 mM sucrose, 30 mM Tris-HCl, protease inhibitor cocktail, phosphatase inhibitor cocktail, pH 7.4), and the concentration measured by BCA method was saved at -80°C.

### 2.14. Intracranial Glioma Xenograft Model

All animal experiments comply with “Guide for the Care and Use of Laboratory Animals” of the National Institutes of Health. U87-luc cells (5 × 10^5^) were injected under the guidance of a stereotaxic instrument to the right frontal lobe of the female BALB/c nude mice (Medical Laboratory Animal Center of Southern Medical University, China) under a general anesthetic. After the operation was completed, the scalp was sutured and returned to the animal room after the animals have awakened. Tumor growth was measured with the IVIS.

### 2.15. Experiment of Glioma Cells Uptake MSC Membranes In Vitro

After extracting the cell membranes, incubate with DiO at RT and protect from light for half an hour, then centrifuge with 25000*g*, 20 mins, 4°C, wash with PBS two times, and obtain DiO-labeled cell membranes stored at 4°C protected from light. Add 10 *μ*l PM (approximately 1/5 of the 10 cm diameter cell culture dish) to each well of the 12-well plate, and then incubate 0.5 h/2 h/4 h/6 h/8 h/10 h, digested cells and centrifuge with 1000 rpm and the pellet wash with PBS. Detection of DiO signals in glioma cells by flow cytometry. PM and glioma cells were incubated together in a confocal dish for 8 hours, fixed with 4% paraformaldehyde, and then blocked with 5% BSA at RT for 1 hour. Then incubated with antibody at RT for 1 hour or overnight at 4°C, followed by incubation with goat anti-rabbit secondary antibody (Alexa Fluor 555, Invitrogen, USA) for 1 h in a humidified, dark environment. The nuclei were stained with DAPI for 15 mins. Images were obtained with a confocal laser microscope (Leica, Germany).

### 2.16. In Vivo Tumor-Homing Assay of MSC Plasma Membrane

Cell membranes were extracted by the above method when the cell fusion degree was 80~90%. DiR-labeled cell membranes were injected into the tail vein of nude mice on the seventh day after planting U87-Luc cells in the brain. The distribution of mesenchymal stem cell membranes was measured with the IVIS, and the fluorescence signals were observed at 0.5 h, 2 h, 6 h, 12 h, 24 h, 48 h, 72 h.

Red blood cell (RBC) membranes were separated and extracted from the peripheral blood of healthy volunteers by the above-mentioned cell membrane extraction method. DiR-labeled cell membranes were injected into the tail vein of nude mice on the seventh day after planting U87-Luc cells in the brain. The distribution of cell membranes was measured with IVIS. The recording time points were the same as above.

### 2.17. Statistical Analysis

Statistical analyses were performed using SPSS IBM 20.0 or GraphPad Prism version 7.0 (GraphPad Software Inc. USA). Data are expressed as mean ± SD, and differences were considered significant at *P* < 0.05. Statistical significance was determined by the t-test or ANOVA.

## 3. Results

### 3.1. Immunophenotype and Directed Differentiation after Immortalization

**Figure 1:**
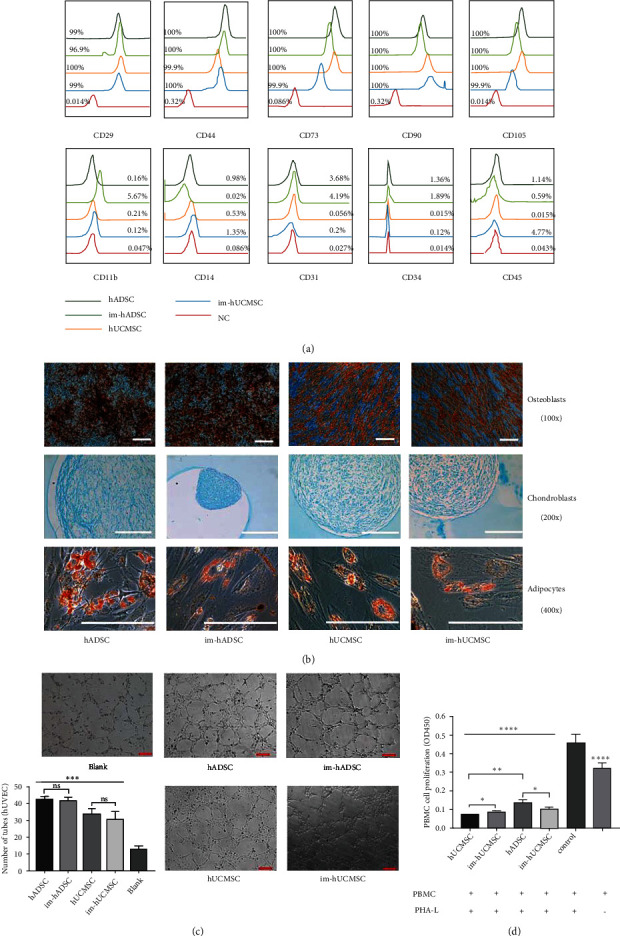
Immunophenotype, directed differentiation and functional properties of MSC and im-MSC. (a) Flow cytometry showing surface markers of mesenchymal stem cells from hADSC and hUCMSC. Immortalization of mesenchymal stem cells did not change the immunophenotype of mesenchymal stem cells. (b) As defined by ISCT, hADSC, hUCMSC, and immortalized mesenchymal stem cells, all have the ability to differentiate into osteoblasts (magnification, ×100), chondroblasts (magnification, ×200), and adipocytes (magnification, ×400). Scale bar, 400 *μ*m. (c) Representative capillary tubule structures were shown for HUVECs treated with culture medium collected from the indicated MSC and im-MSC. magnification, ×200, scale bar, 100 *μ*m. (d) Proliferation of PBMC co-cultured with MSC from different sources treated with mitomycin C. hADSC: human adipose-derived mesenchymal stem cells; hUCMSC: human umbilical cord mesenchymal stem cells; ISCT: International Society for Cell & Gene Therapy. ∗*P* < 0.05, ∗∗*P* < 0.01, ∗∗∗*P* < 0.001, ∗∗∗∗*P* < 0.0001. ns: not significant.

The International Society for Cell & Gene Therapy (ISCT) proposes the following minimal criteria to define human MSC [20, 21]: 1) MSCs must be plastic-adherent when maintained in standard culture conditions; 2) MSCs must express CD105, CD73, and CD90 and not express CD45, CD34, CD14, CD11b, CD79a, CD19, and HLA-DR surface molecules; and 3) MSCs must differentiate into osteoblasts, adipocytes, and chondroblasts in vitro. In our current study, we extracted hUCMSCs from umbilical cord tissue (Figure S1A), and flow cytometry showed that im-MSCs and unengineered MSCs all maintained the MSC immunophenotype proposed by the ISCT. High expression of CD105, CD73, and CD90 were detected in primary MSCs (Figure 1(a)). In particular, long-term cultured im-hADSCs (P55) and im-hUCMSCs (P30) expressed high levels of CD105, CD70, and CD90. Simultaneously, all MSCs lacked the expression of CD45, CD34, CD14, or CD11b (Figure 1(a)). Engineered MSCs maintained the ability to differentiate into mesenchymal lineages, forming osteoblasts (Alizarin Red staining), chondroblasts (Alcian blue staining), and adipocytes (Oil Red O staining) in vitro (Figure 1(b)). These results indicate that im-MSCs conform to the basic definition of MSCs as proposed by the ISCT.

### 3.2. Functional Properties of Im-MSCs

To evaluate the pro-angiogenic ability of im-MSCs, HUVECs were treated with different types of conditioned medium (CM), and then the formed endothelial cell tubes were then visualized (Figure 1(c)). Compared with the control group, the CM of MSCs and im-MSCs from different sources could significantly (*P* < 0.0001) promote the tube-forming ability of HUVEC in vitro. Specifically, hADSCs and im-hADSCs could better enhance the tube-forming ability of HUVECs than hUCMSCs and im-hUCMSCs. However, no significant difference was found between im-MSCs and MSCs from the same source.

We further studied the effect of im-MSCs on the proliferation ability of PHA-L activated PBMC to evaluate the immunosuppressive ability of im-MSCs (Figure 1(d)). The results show that compared with the control group, both MSCs and im-MSCs had stronger ability to inhibit the proliferation of immune cells. Interestingly, MSCs of the same origin also exhibit different immunosuppressive abilities before and after immortalization. Specifically, hUCMSCs had stronger immunosuppressive ability than im-hUCMSCs, but the opposite result was obtained in hADSCs. There were also certain differences between MSCs from different sources. hUCMSCs can inhibit the proliferation of activated PBMC better than hADSCs. The two types of im-MSCs showed no difference in their ability to inhibit the proliferation of PBMC.

### 3.3. Expression of the Immortalizing Genes

**Figure 2:**
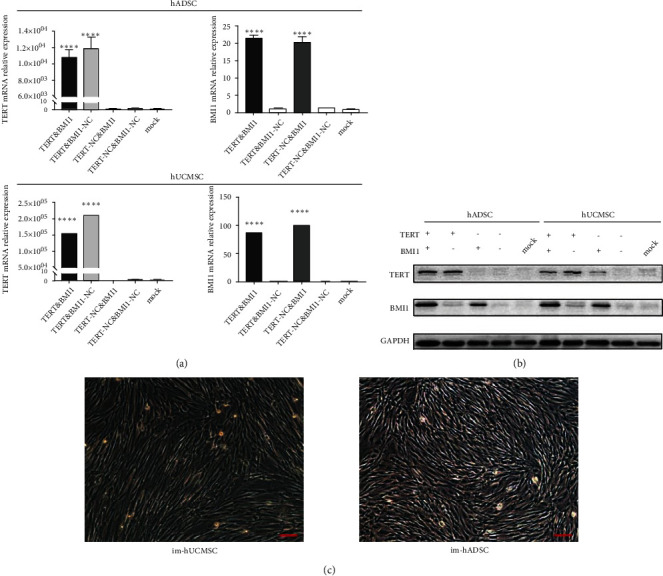
Protein and mRNA expression of BMI1 and TERT genes in immortalized MSCs. (a) RT-PCR analysis of BMI1 and TERT mRNA expression in hADSC and hUCMSC infected with LV-TERT, LV-BMI1, and control lentivirus (mean ± SD, *n* = 3). (b) Western blot detection of TERT and BMI1 protein expression in hADSC and hUCMSC after overexpressing TERT or BMI1. (c) Microscopic view of immortalized hADSC and hUCMSC, respectively, magnification, ×100, scale bar, 100 *μ*m. The results are presented as the mean ± SD of biological triplicate assays. ∗∗∗∗*P* < 0.0001. LV: lentivirus.

RT-qPCR and western blotting confirmed the efficient introduction of the immortalizing genes. Engineered MSCs highly expressed TERT and BMI1 mRNA and protein relative to primary MSCs (Figures 2(a) and 2(b)). Cells morphology did not change after engineering and showed fibroblast-like adherent growth (Figure 2(c)).

### 3.4. Cell Proliferation and Population Doubling Levels

**Figure 3:**
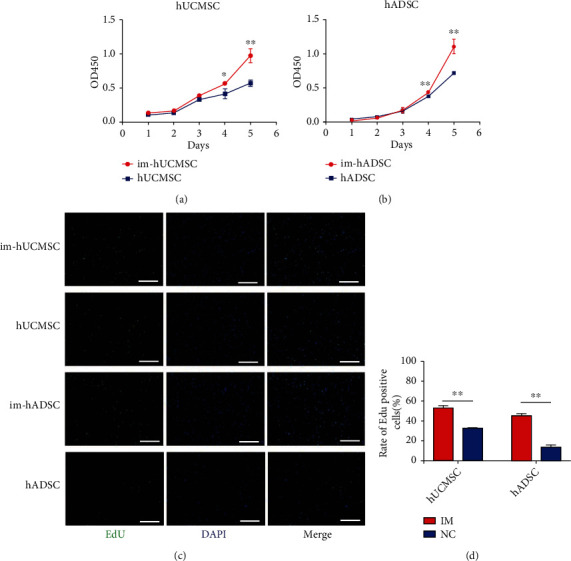
Immortalized mesenchymal stem cells have greater proliferative capacity. (a) and (b) Cell growth curve of hADSC, hUCMSC, and MSCs after transfected by LV-TERT and LV-BMI1, detected by the CCK-8 assay. (c) and (d) By adding EdU reagent, the EdU-positive cells of hADSC, hUCMSC im-hUCMSC, and im-hADSC were detected at two hours later. The rate of EdU-positive cells in hADSC, hUCMSC, im-hUCMSC, and im-hADSC. The results are presented as the mean ± SD of biological triplicate assays. Magnification, ×100. Scale bar, 500 *μ*m. ∗*P* < 0.05, ∗∗*P* < 0.01. im-hUCMSC: immortalized human umbilical cord mesenchymal stem cells; im-hADSC: immortalized human adipose-derived mesenchymal stem cells.

The proliferation of engineered cells allowed us to observe the biological processes of im-MSCs transfected with LV-TERT and LV-BMI1. The absorbance measured at 450 nm represents cell growth and was recorded by a microplate reader. The cell growth curve increased significantly (*P* < 0.01) four days after transfection (Figures 3(a) and 3(b)). Cell proliferation was detected using an EdU incorporation assay, and the rate of EdU-positive cells was significantly (*P* < 0.01) increased after immortalization (Figures 3(c) and 3(d)). It was observed that im-MSCs had a higher proliferative capacity than non-im-MSCs. Additionally, im-hADSCs could be cultured for more than population doubling levels (PDLs) 60 (Figure S2A, B) and Im-hUCMSCs could be cultured for more than PDLs 30 (Figure S2C, D). After resuscitating the frozen immortalized MSCs, the cells maintained their original fibroblast-like morphology. When the degree of cell fusion reaches more than 80%, the cells can be seen spirally arranged.

### 3.5. Genetic Stability

**Figure 4:**
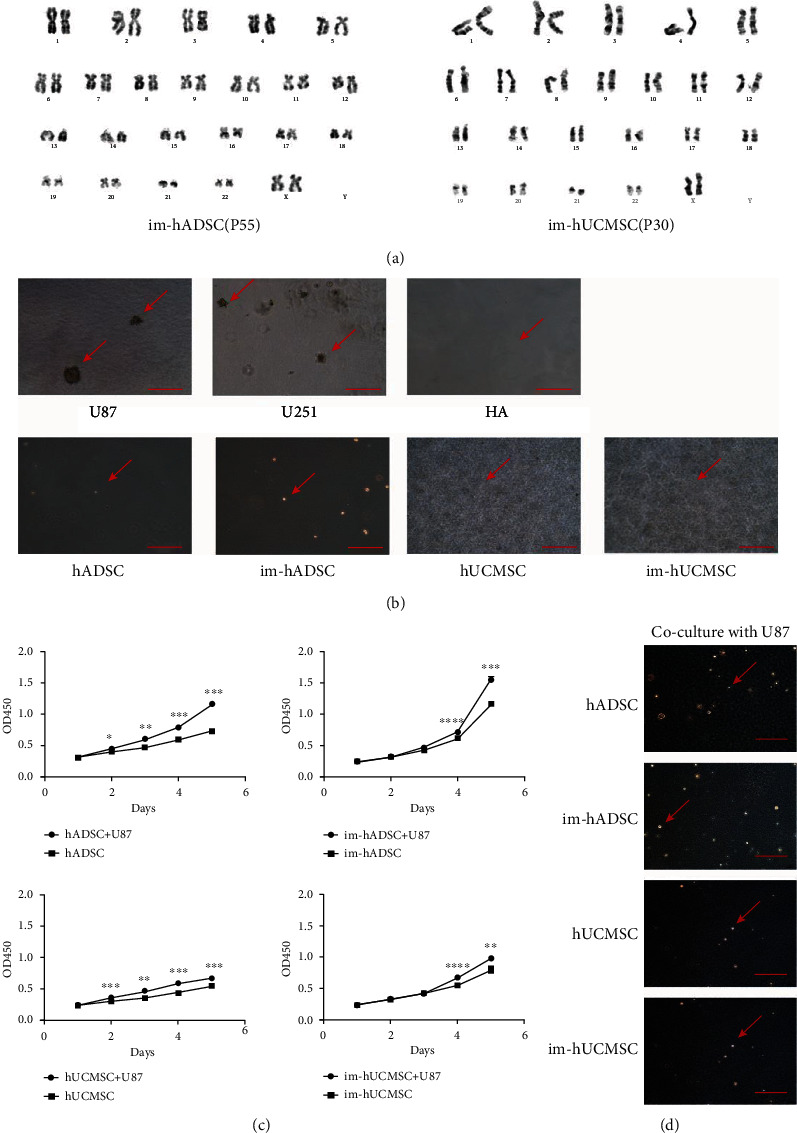
Safety of immortalized mesenchymal stem cells in vitro. (a) Karyotype analysis of immortalized mesenchymal stem cells. (c) Soft agar clone formation test to detect the clone formation ability of immortalized mesenchymal stem cells, GBM cell lines (U87 and U251) were used as a positive reference, and normal glial cells (HA1800) were used as a negative reference. (c) Cell proliferation curve of mesenchymal stem cells that indirectly co-cultured with U87 cells, detected by the CCK-8 assay. (d) Soft agar clone formation experiment was used to detect the clone formation ability of immortalized mesenchymal stem cells co-cultured with U87 cells indirectly. Magnification, ×100. Scale bar, 500 *μ*m. ∗*P* < 0.05, ∗∗*P* < 0.01, ∗∗∗*P* < 0.001, ∗∗∗∗*P* < 0.0001.

To determine the genetic stability of engineered MSCs, we performed karyotyping on colchicine-treated im-MSCs after long-term culture. No significant genetic instability was found in the im-hADSC (P55) or im-hUCMSC (P30) (Figure 4(a)).

### 3.6. Safety of Im-MSCs In Vitro and In Vivo

Soft agar (1.2%) containing double medium was spread on the bottom of the 6-well plate. U87, U251, HA1800, hUCMSC, hADSC, im-hUCMSC, and im-hADSC were mixed separately in 0.7% soft agar with double medium, and the mixture was spread on 1.2% soft agar. The mixture was allowed to solidify, and the 6-well plate was placed at 37°C in a humidified incubator with 5% CO_2_. U87 and U251 were negative references and HA1800 was a positive reference. After three weeks, the U87 and U251 (Figure 4(b)) groups showed formation of cell-cloned spheres under the microscope, whereas the HA1800 negative reference group showed no sphere formation (Figure 4(b)). No clones were formed in either hUCMSCs, hADSCs, im-hUCMSCs, or im-hADSCs (Figure 4(b)). Therefore, we believe that im-hUCMSCs and im-hADSCs were not tumorigenic in vitro.

**Figure 5:**
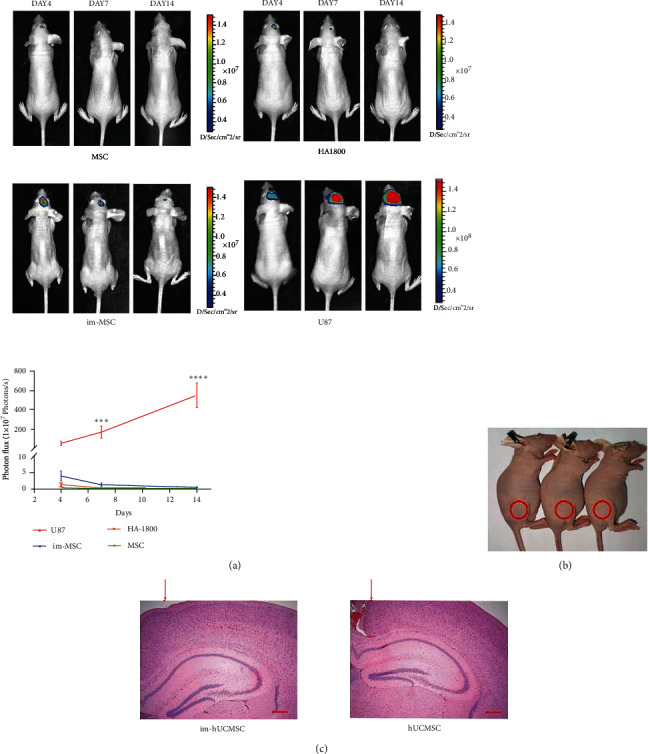
Safety of immortalized mesenchymal stem cells in vivo. (a) IVIS luminescent imaging of BALB/c nude mice with intracranial injection of immortalized umbilical cord mesenchymal stem cells (1 × 10^6^). Line graph showing cell growth in the brain, U87 cells were used as a positive reference, and normal glial cells (HA1800) were used as a negative reference. (b) Im-hUCMSC were injected subcutaneously into the proximal right lower extremity of nude mice to evaluate tumorigenic; the picture was taken on the 14th day after injection. (c) HE staining shows that there was no abnormal tissue growth inside the brain where mesenchymal stem cells were injected. Red arrow indicates the direction of needle entry. Magnification, ×50. Scale bar, 500 *μ*m. ∗∗∗*P* < 0.001, ∗∗∗∗*P* < 0.0001. IVIS: in vivo imaging system; HE: hematoxylin-eosin.

Next, Im-hUCMSCs were transduced with lentiviral constructs pLenti-CMV-mCherry-linker-Luc-PGK-Blasticidin, sorted, and screened for mCherry fluorescent protein and firefly luciferase (Fluc) expression. Simultaneously, HA1800 cells were transduced with LV-mCherry-Luc as negative cells. U87-Luc was constructed as a positive reference (Figure S3). The engineered cells were injected into the cranium of female BALB/c nude mice (*n* = 5). The growth of the cells in the body was observed using the IVIS after intraperitoneal injection of D-Luciferin potassium salt (150 *μ*g/g, PerkinElmer, Waltham, Massachusetts, U.S.A.) for 10 mins. The growth of the graft in the brain of mice was recorded on the 4th, 7th, and 14th day after implantation (Figure 5(a)). U87-Luc grew in the brain, eventually leading to the death of the mice. However, HA1800, hUCMSC, and im-hUCMSC slowly disappeared after a period of survival in the brain (Figure 5(a)). Two weeks after the injection of immortalized hUCMSCs under the skin of mice, no abnormal tissues were found (Figure 5(b)). There were no abnormal tissues in the brain planting site even after HE staining (Figure 5(c)).

### 3.7. Co-Cultivation of U87 and MSC

The indirect co-incubation of U87 and MSCs in 0.4-*μ*m Transwell plates for 48 h enhanced the proliferation capacity of MSCs (Figure 4(c)). However, the co-cultured MSCs could not form clonal spheres in the soft agar colony formation assay (Figure 4(d)).

### 3.8. Glioma Cells Can Easily Take up Im-MSC Membranes without Affecting Their Proliferation In Vitro

**Figure 6:**
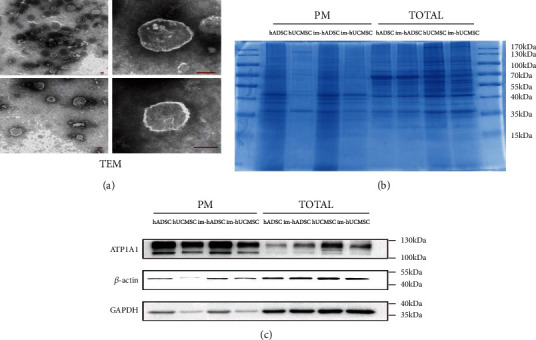
Extraction of mesenchymal stem cell membrane. (a) TEM image of mesenchymal stem cell membranes under electron microscope. (b) The expression profile of proteins in PM and whole-cell lysates of MSCs or im-MSCs were analyzed using SDS-PAGE. (c) The expression levels of ATP1A1, *β*-actin, and GAPDH in PM and whole-cell lysates were evaluated using western blot. Scale bar, 100 nm. TEM: transmission electron microscope; PM: plasma membrane.

**Figure 7:**
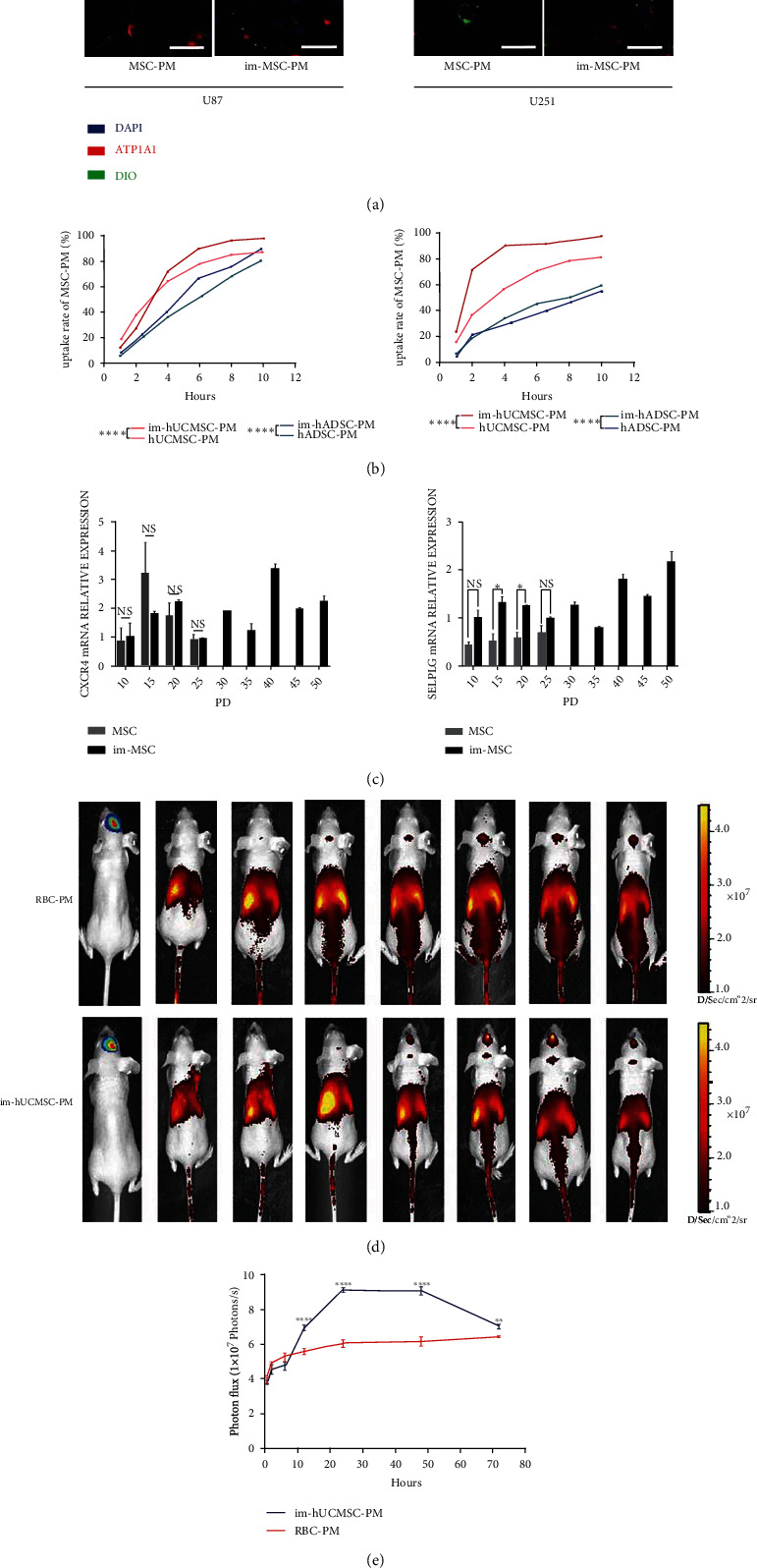
(a)–(c) Cellular uptake of PM and expression of tumor-homing molecules. (d)–(e) Biodistribution of im-MSCs PM after intravenous (IV) injections. (a) IF staining of ATP1A1 (red) in U87 and U251, PM marked with DIO (green), magnification, ×200. Scale bar, 50 *μ*m. (b) Flow cytometry showing the uptake curve of MSC-PM by U87 and U251. (c) Real-time reverse transcription-PCR (RT-PCR) detection of CXCR4 and SELPLG mRNA expression in hADSCs and im-hADSCs of different PDs. The results are presented as the mean ± SD of biological triplicate assays. ∗*P* < 0.05. (d) Representative IVIS luminescence imaging at different time points (0.5 hours, 2 hours, 6 hours, 12 hours, 24 hours, 48 hours, and 72 hours) after mice bearing gliomas were injected with cell membranes (im-hUCMSC-PM or RBC-PM). (e) The fluorescence signal intensity of the mice. The results are presented as the mean ± SD of biological triplicate assays. ∗*P* < 0.05, ∗∗*P* < 0.01, ∗∗∗∗*P* < 0.0001. ns: not significant. IF: immunofluorescence; PD: population doubling.

We studied the potential of im-MSC membranes as drug carriers for treating glioma by extracting and observing the cell membranes of diameters approximately 100–150 nm under an electron microscope (Figure 6(a)); the dimension assisted passage through the blood–brain barrier. SDS-PAGE gel analysis revealed that the protein profiles of the plasma membranes were distinct from those of whole-cell lysates (Figure 6(b)). At the same protein concentration, the plasma membrane contained more ATP1A1 protein and less *β*-actin and GAPDH. The former was located on the cell membrane, whereas the latter two proteins were in the cytoplasm (Figure 6(c)). The key factor in the cell membrane drug delivery system is the ability to be taken up by tumor cells. After the DiO-labeled cell membrane was added to the medium, the cell membrane was ingested by glioma cells, as detected by confocal microscopy and flow cytometry (Figures 7(a) and 7(b)).

### 3.9. Expression of Homing Molecules Maintained by Im-MSCs on the Cell Membranes

We extracted RNA from every five generations of MSCs and im-MSCs during long-term culture to detect the long-term expression of homing-related molecules. We detected the expression of homing molecules by qPCR. Molecules associated with MSC homing included the chemokine receptor CXCR4 and adhesion ligands, such as PSGL-1 (SELPLG). Membrane proteins were the main determiners of MSC homing behavior. In MSCs, the expression of these molecules after long-term culture was maintained, as detected by qPCR (Figure 7(c)). Interestingly, the expression level of SELPLG in im-MSCs was higher than that in unengineered MSCs in some PDLs. Therefore, long-term stable expression of homing molecules supports im-MSCs as cellular or cell membrane-based drug carriers.

### 3.10. Tumor Tropism with Respect to In Site Glioma Retained by Im-MSC Membranes In Vivo

MSC membranes were extracted and labeled with DiR and injected into tumor-bearing mice through the tail vein to detect the in vivo targeting ability of MSC membranes to glioma. The fluorescence signals were observed at 0.5, 2, 6, 12, 24, 48, and 72 h (Figure 7(d)). The accumulation peaked 24-48 h after administration and then decreased gradually (Figure 7(e)). We used DiR-labeled RBC-PM as a control group and injected it into tumor-bearing mice through the tail vein to prove the superiority of im-MSCs in tumor homing. The results showed that the cell membrane of im-MSCs had better glioma homing ability at 12 h/24 h/48 h/72 h after injection compared with RBC cell membrane lacking homing molecules on the membrane surface. (Figure 7(e)). Immortalized cell membranes can be used as drug carriers to help the drugs reach and accumulate in the glioma, thereby exerting therapeutic effects.

## 4. Discussion

CAR-T cell therapy opened new fields for the treatment of tumors. CAR-T therapy is often used in cancers of the blood system; however, for solid tumors [22, 23], especially gliomas [24], it is not effective and a new method of cell therapy needs to be discovered. Stem cells have received increasing attention owing to their unique natural homing ability. Several clinical trials of MSCs have been approved, but the long-term safety of MSCs remains a concern. Researchers have constructed engineered MSCs that contain a suicide system based on an inducible caspase-9 protein [25]. This system can direct MSC killing to provide the necessary safety by a chemical inducer of dimerization in vivo. However, the system requires a complicated construction process, and because of the limited passage capacity of MSC, the system needs to be repeatedly constructed. The above factors limit the application of the system. In the application of MSCs to the treatment of tumors, one of the most serious problems is the tumorigenicity of MSCs, especially the im-MSCs constructed by lentivirus transfection, which has a stronger proliferative capacity than ordinary MSCs. However, the greater in vivo viability of im-MSCs allows them to secrete anti-tumor substances longer. In theory, a longer retention time of cellular anti-tumor agents means longer lasting effects and longer dosing cycles [26]. Based on this point, im-MSCs are an attractive choice for cancer treatment, but their application is based on the premise that it is safe. In our study, we observed that im-MSCs do not have tumorigenicity either in vivo or in vitro. In particular, we investigated its safety as a carrier for gliomas. The potential tumorigenicity of im-MSCs implicates them as cell sources for long-term cell therapy, especially for diseases that require long-term maintenance treatment.

Immortalization provides more cells, but the use of a lentivirus as an engineering tool may hinder its clinical translation. The use of other gene transfer pathways in future studies (such as electroporation), or non-genetic pathways (such as repeated transfection of recombinant proteins containing cell-penetrating peptides), may allow their clinical application. Although im-MSCs are not tumorigenic, their biological behavior in vivo, especially in the tumor microenvironment and their interactions with tumor cells, still needs to be considered. In short, MSCs still pose many problems as whole-cell drug carriers, and an effective method in the future should be to screen out a subset of MSCs with anti-tumor effects and immortalize them as whole-cell drug carriers. This way, the adverse effects caused by the heterogeneity of MSCs can be avoided. Screening and immortalizing anti-tumor subgroups could boost the production of synergistic anti-tumor effects with drugs by anti-tumor subsets as far as possible. Another strategy is to use stem cell products to load drugs to exert anti-tumor effects.

Other natural drug carriers, such as cell membranes and extracellular vesicles (EVs), have advantages as drug carriers [27] as they have better biocompatibility and biodegradability than chemically synthesized drug carriers [28]. Natural materials have lower toxicity and immunogenicity than chemically synthesized nanocarriers [29]. The types of drugs delivered by EVs mainly include siRNA, miRNA, and small-molecule compounds. Glioblastoma-bearing mice have been treated with exosomes derived from MSCs with high expression of miR-146b, which has anti-cancer effects [30]. The same strategy uses MSC-derived EVs to deliver inhibitors of miRNA that promote cancer, thereby inhibiting tumor progression, such as miR-9 [31]. EVs were injected with a small-molecule drug, JSI-124, into a mouse model of glioblastoma and it showed tumor-suppressive effects [32]. However, EVs have low acquisition rates, low drug loading, and time-consuming preparation processes, which limit their application in the clinic. Alternatively, im-MSCs provide abundant exosome sources for clinical application because of their strong proliferation ability and large passage number. In particular, after immortalizing MSCs that inhibit tumors, they can be used as a source of cells for exosomal vectors, which may achieve better anti-tumor effects than exosomes from unfiltered MSCs. Cell membranes can be an alternative source of EVs and serve as carriers for anti-tumor drugs because of their easy acquisition and less time-consuming preparation processes. At the molecular level, MSC homing involves chemokine receptors CCR2, CXCL12, and CXCR4 and adhesion ligands, such as PSGL-1 and SLeX [14]. Among these molecules, CXCR4 and SELPLG play a key role in the tumor-tropic processes of MSCs. The sustained expression of these molecules is essential for the use of complete MSCs and MSC membranes as therapeutic carriers. Moreover, most of these molecules are membrane proteins, which further indicates the potential of MSC membranes as therapeutic carriers. In our research, the immortalized cells were observed to maintain the continuous expression of these key molecules, which also provides the molecular basis for the use of immortalized mesenchymal stem cell membranes for tumor homing. Interestingly, im-hMSC showed a stronger ability to be taken up by glioma cells in the uptake experiment. This phenomenon may be related to the greater expression of homing molecules by im-MSCs in certain periods. Im-MSC-PM showed stronger homing ability than red blood cell membranes in vivo. This may also be attributed to the expression of the im-MSC homing molecule. At the same time, it can be seen from the experimental results that RBC-PM also shows accumulation in the tumor site over time. This may be due to the long circulation time of RBCs, the EPR (enhanced permeability and retention) effect, and the high blood flow at the tumor site.

## 5. Conclusion

Immortalized MSCs maintain the immunophenotype and induce the differentiation of MSCs. Engineered MSCs also retain the functional characteristics of MSCs specified by ISCT, especially the immunosuppressive ability and the ability to promote angiogenesis. The im-MSCs showed no tumorigenicity in vitro or in vivo, especially when injected directly into the brain, and no tumor formation was observed; thus, im-MSCs can be used as safe cell sources for MSC-related tumor treatment. Moreover, the cell membranes of im-MSCs showed a homing ability to gliomas; therefore, im-MSC membranes are a potential candidate for the natural carrier of drug delivery. They can be used as drug carriers for the treatment of glioma by tumor homing. In the future, im-MSC-based regimens may augment current treatment modalities in glioma.

## Data Availability

Data sharing not applicable to this article as no datasets were generated or analyzed during the current study.

## Supplementary Materials

Supplementary Materials Figure S1 Isolation and extraction of hUCMSCs from human umbilical cord tissue. Mesenchymal stem cells crawl out of the gel after adherent tissue culture for 2-3 weeks. Picture shows the growth of the first generation of umbilical cord mesenchymal stem cells. Scale bar, 500 *μ*m. Figure S2 Continuous culture of mesenchymal stem cells in vitro. (A) hADSCs cultured to PDLs 28 in vitro. (B) Continuous culture of hADSCs after immortalization in vitro, PDLs >60. PDLs 60 not shown. (C–D) Culture of hUCMSCs and im-hUCMSCs. Scale bar, 500 *μ*m. (E) Population doubling levels of MSC and im-MSC. ∗∗∗*P* < 0.001. (F) Phase-contrast microscopy showing the state of im-MSC before freezing and after rewarming. Magnification, ×100, scale bar, 500 *μ*m. Figure S3 Construction of engineered cells. U87 and im-MSC were infected by LV (pLenti-CMV-mCherry-linker-Luc-PGK-Blasticidin) express mCherry fluorescent protein. Scale bar, 500 *μ*m. PDLs, population doubling levels. Supplementary Table 1 Primers sequences of RT-PCR. Supplementary Table 2 Antibodies used in this study.
