# Supplementary material for: Immortalized Mesenchymal Stem Cells: A Safe Cell Source for Cellular or Cell Membrane-Based Treatment of Glioma
Source: Stem Cells Int. 2022 Apr 13;2022:6430565. doi: 10.1155/2022/6430565 (PMC9020902; doi:10.1155/2022/6430565)
Supplement: Supplementary Materials — Figure S1 Isolation and extraction of hUCMSCs from human umbilical cord tissue. Mesenchymal stem cells crawl out of the gel after adherent tissue culture for 2-3 weeks. Picture shows the growth of the first generation of umbilical cord mesenchymal stem cells. Scale bar, 500 μm. Figure S2 Continuous culture of mesenchymal stem cells in vitro. (A) hADSCs cultured to PDLs 28 in vitro. (B) Continuous culture of hADSCs after immortalization in vitro, PDLs >60. PDLs 60 not shown. (C–D) Culture of hUCMSCs and im-hUCMSCs. Scale bar, 500 μm. (E) Population doubling levels of MSC and im-MSC. ∗∗∗P < 0.001. (F) Phase-contrast microscopy showing the state of im-MSC before freezing and after rewarming. Magnification, ×100, scale bar, 500 μm. Figure S3 Construction of engineered cells. U87 and im-MSC were infected by LV (pLenti-CMV-mCherry-linker-Luc-PGK-Blasticidin) express mCherry fluorescent protein. Scale bar, 500 μm. PDLs, population doubling levels. Supplementary Table 1 Primers sequences of RT-PCR. Supplementary Table 2 Antibodies used in this study. [file 6430565.f1.pdf]

Supporting Information

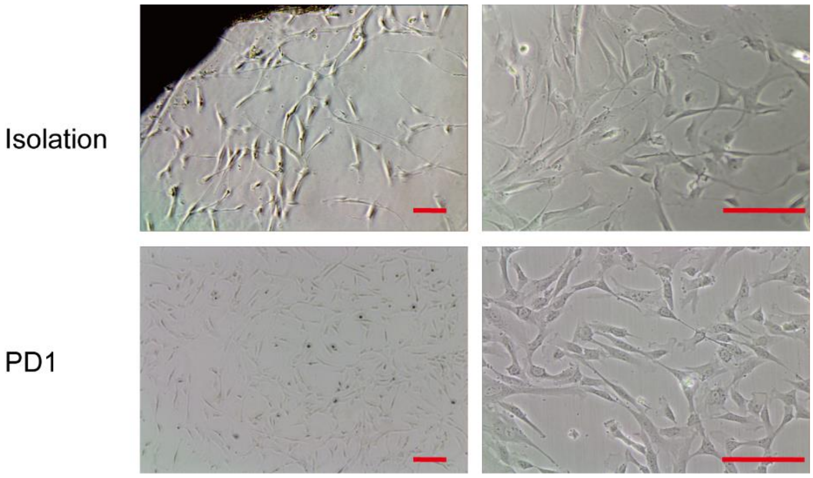

Fig. S1 Isolation and extraction of hUCMSCs from human umbilical cord tissue. Mesenchymal stem cells crawl out of the gel after adherent tissue culture for 2-3 weeks. Picture shows the growth of the first generation of umbilical cord mesenchymal stem cells. Scale bar, 500 μm.

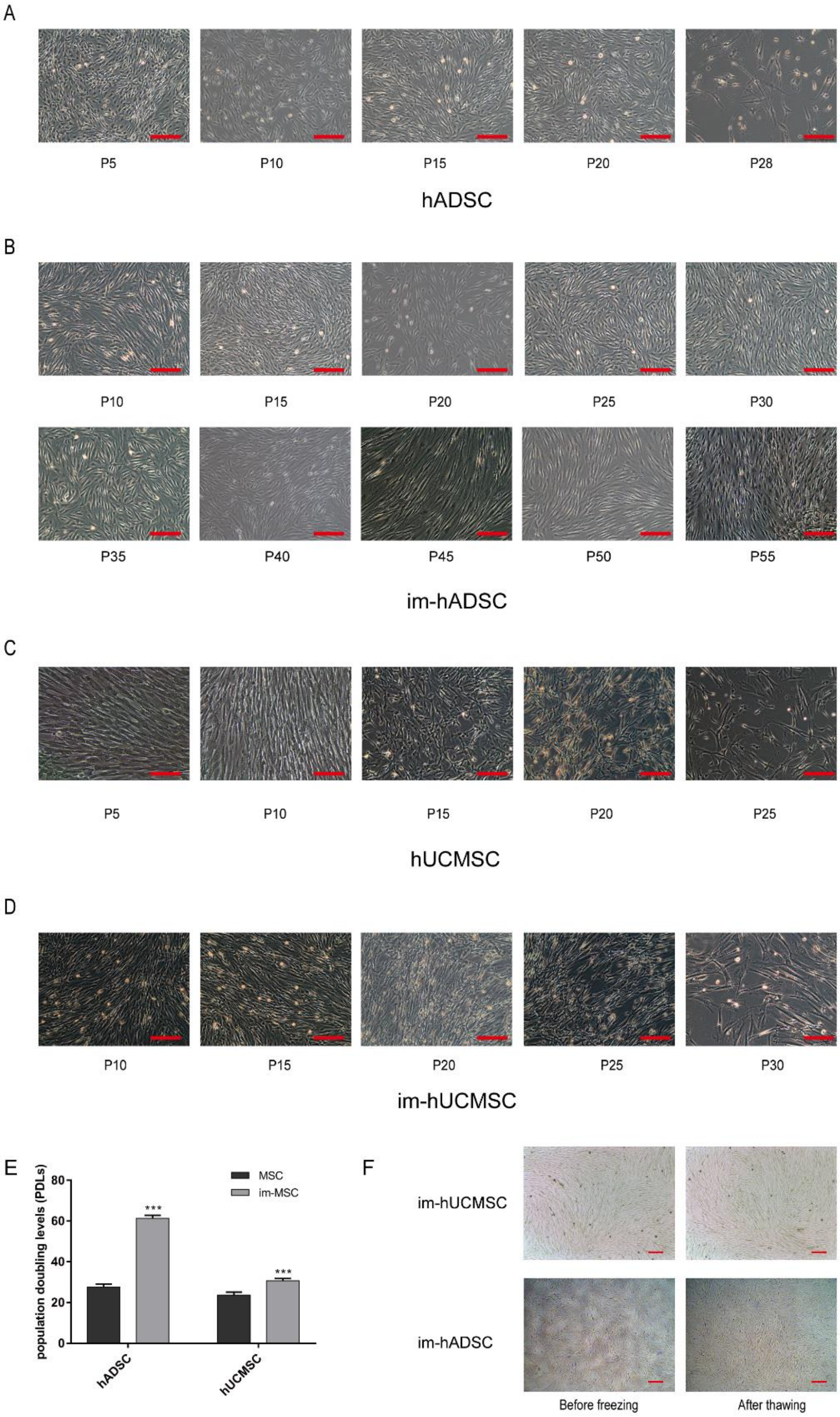

Fig. S2 Continuous culture of mesenchymal stem cells in vitro. (A) hADSCs cultured to PDLs 28 in vitro. (B) Continuous culture of hADSCs after immortalization in vitro, PDLs > 60. PDLs 60 not shown. (C-D) Culture of

hUCMSCs and im-hUCMSCs. Scale bar, 500 μm. (E) Population doubling levels of MSC and im-MSC. ***P < 0.001. (F) Phase-contrast microscopy showing the state of im-MSC before freezing and after rewarming. Magnification, ×100, Scale bar, 500 μm.

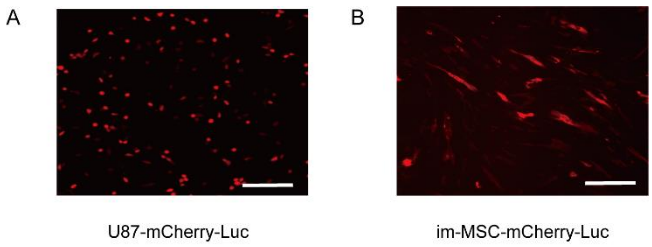

Fig. S3 Construction of engineered cells. U87 and im-MSC cells were infected by LV (pLenti-CMV-mCherry-linker-Luc-PGK-Blasticidin) express mCherry fluorescent protein. Scale bar, 500 μm. PDLs, population doubling levels.

Supplementary Table 1

Primers sequences of RT-PCR.

Gene	Forward Primer	Reverse Primer
TERT	AAATGCGGCCCCTGTTTCT	CAGTGCGTCTTGAGGAGCA
BMI1	CCACCTGATGTGTGTGCTTTG	TTCAGTAGTGGTCTGGTCTTGT
CXCR4	TACACCGAGGAAATGGGCTCA	AGATGATGGAGTAGATGGTGGG
SELPLG	TGTTGCTGATCCTACTGGGC	CACAGTGGTAGACTCAGGGGT
GAPDH	GGCACCGTCAAGGCTGAGAAC	GGTGGCAGTGATGGCATGGAC

Supplementary Table 2

Antibodies used in this study

Antibodies	Manufacturer	Catalog No.	Application
CD11b	BD	555388	FC
CD14	BD	555399	FC
CD29	BD	557332	FC
CD31	BD	555445	FC
CD34	BD	555822	FC
CD44	BD	555478	FC
CD45	BD	555482	FC
CD73	BD	560847	FC
CD90	BD	555596	FC
CD105	BD	561443	FC
TERT	Solarbio	K003548P	WB
BMI1	BOSTER	PB0102-0.1	WB
ATP1A1	Proteintech	14418-1-AP	WB, IF
β-ACTIN	Proteintech	60008-1-Ig	WB
GAPDH	Proteintech	60004-1-Ig	WB

FC: Flow cytometry; WB: Western blot; IF: Immunofluorescence
